# Left-right asymmetric expression of *dpp* in the mantle of gastropods correlates with asymmetric shell coiling

**DOI:** 10.1186/2041-9139-4-15

**Published:** 2013-05-28

**Authors:** Keisuke Shimizu, Minoru Iijima, Davin HE Setiamarga, Isao Sarashina, Tetsuhiro Kudoh, Takahiro Asami, Edi Gittenberger, Kazuyoshi Endo

**Affiliations:** 1 Department of Earth & Planetary Science, The University of Tokyo, 7-3-1 Hongo, Tokyo, 113-0033, Japan; 2 Graduate School of Life and Environmental Sciences, University of Tsukuba, 1-1-1 Tennodai, Tsukuba, 305-0006, Japan; 3 College of Life and Environmental Sciences, University of Exeter, Stocker Road, Exeter, EX4 4QD, UK; 4 Department of Biology, Shinshu University, Mastumoto, 390-0802, Japan; 5 Netherlands Center for Biodiversity, Leiden, The Netherlands

**Keywords:** Left-right asymmetry, *Decapentaplegic*, Shell coiling, Gastropods

## Abstract

**Background:**

Various shapes of gastropod shells have evolved ever since the Cambrian. Although theoretical analyses of morphogenesis exist, the molecular basis of shell development remains unclear. We compared expression patterns of the *decapentaplegic* (*dpp*) gene in the shell gland and mantle tissues at various developmental stages between coiled-shell and non-coiled-shell gastropods.

**Results:**

We analyzed the expression patterns of *dpp* for the two limpets *Patella vulgata* and *Nipponacmea fuscoviridis*, and for the dextral wild-type and sinistral mutant lineage of the pond snail *Lymnaea stagnalis*. The limpets had symmetric expression patterns of *dpp* throughout ontogeny, whereas in the pond snail, the results indicated asymmetric and mirror image patterns between the dextral and sinistral lineages.

**Conclusion:**

We hypothesize that Dpp induces mantle expansion, and the presence of a left/right asymmetric gradient of the Dpp protein causes the formation of a coiled shell. Our results provide a molecular explanation for shell, coiling including new insights into expression patterns in post-embryonic development, which should aid in understanding how various shell shapes are formed and have evolved in the gastropods.

## Background

**Figure 1:**
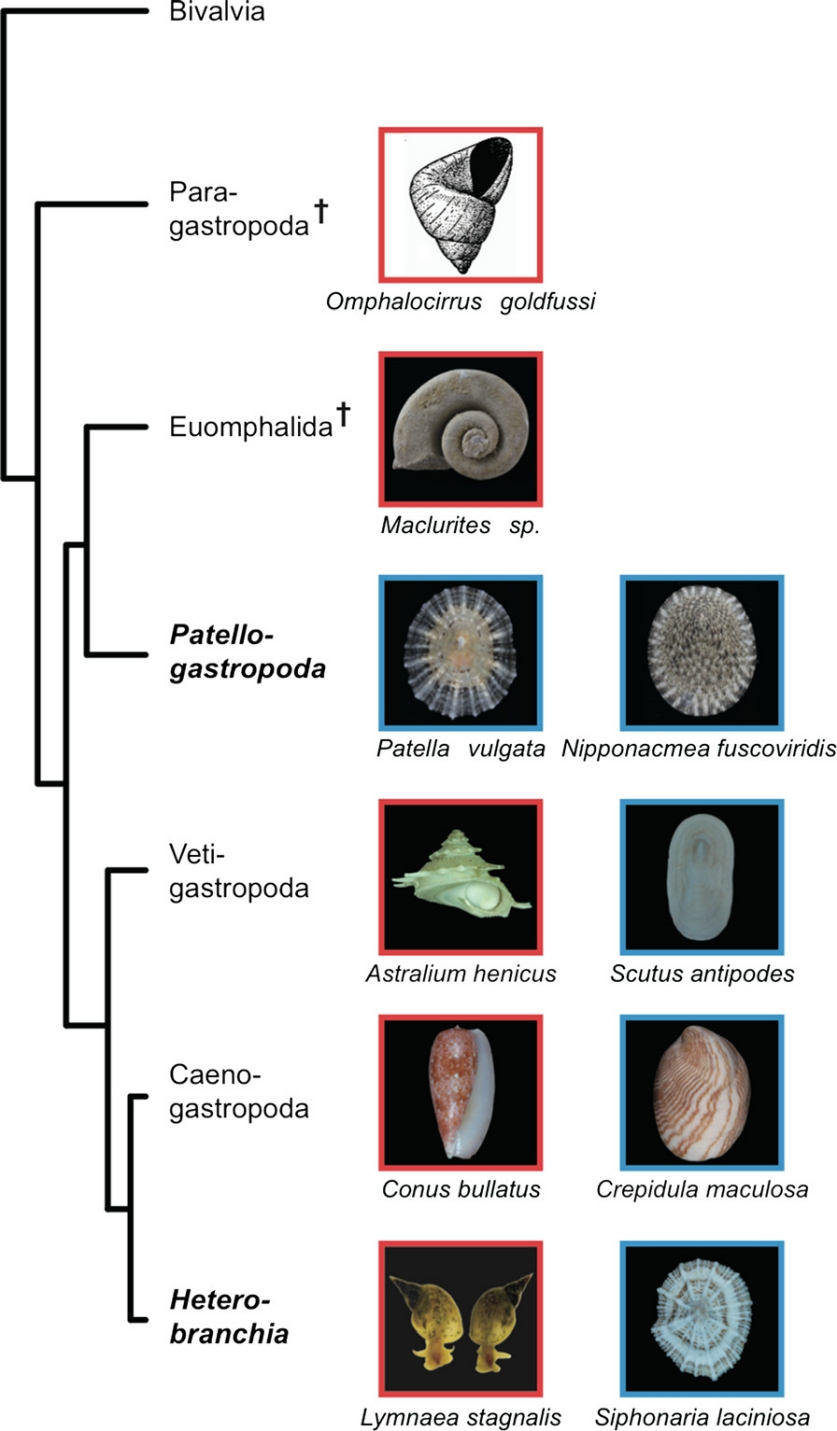
**Phylogeny of the Gastropoda and major shell shapes in each group.** The phylogeny is based on the studies of Ponder *et al*. [1] and Aktipis *et al*. [2]. Red boxes indicate coiled shell and blue boxes indicate non-coiled shell. Dagger symbols indicate extinct taxa. Illustration of Paragastropoda is from Knight *et al*. [3].

Gastropoda is arguably the most diverse molluscan group. Its members have adapted to various marine and terrestrial ecological niches. One of their distinguishing features is the presence of an external shell in most species. Typologically, the shells can be classified into two groups, coiled and non-coiled (Figure 1). Such a general grouping, however, is highly arbitrary because both groups are likely to be non-monophyletic. Recent phylogenetic and paleontological studies suggest the possibility that shell coiling evolved at the base of the Gastropoda lineage, and that secondary losses of shell coiling occurred several times in various lineages (Figure 1) [1,2]. However, although the possible evolutionary path of shell coiling can be inferred from phylogenetic studies, the mechanistic explanation of the morphological changes is not yet understood.

To understand the origin of such morphological diversity, we need to look at the developmental mechanisms of the shells. The developmental process of gastropod shells has already been described [4,5]. The shell gland is formed by the invagination of ectodermal cells at the early trochophore stage [4]. In the trochophore, shell-secreting cells in the shell gland start to form the initial shell. The mantle tissue begins to develop at the veliger stage, and takes over the role of shell secretion for most of the organism’s life [5]. Thus, the shell gland is important in early shell formation, when the initial trigger and early processes of shell formation occur. Meanwhile, the mantle is involved in shell growth during and after the veliger stage. Accordingly, some previous studies of shell development have focused on these two ‘tissues’.

Despite existence of some studies on gastropod shell formation, molecular embryological insight into shell development remains meager. Nederbragt *et al*. and Iijima *et al*. [6,7] reported that the *decapentaplegic* (*dpp*) gene is expressed around the shell gland, suggesting involvement of *dpp* in shell formation. These studies were not conclusive, however, because they studied *dpp* only in the early stages of embryonic development (late gastrula and trochophore stages). To remedy such lack of information, and to conclusively show if *dpp* is involved in shell development in gastropods, we checked the expression patterns of *dpp* in the later developmental stages in three gastropod species: two limpets with a non-coiling shell (*Patella vulgata* and *Nipponacmea fuscoviridis*) and a pond snail with a coiled shell (*Lymnaea stagnalis*). Because in previous studies, *dpp* expression patterns in early developmental stages up to the trochophore were reported in these three species [6-8], we confirmed the expression patterns in the veligers and adults. To understand the involvement of *dpp* expression in shell coiling, we confirmed the *dpp* expression pattern in the trochophore, veliger, and adults of the sinistral mutant of *L. stagnalis*, which have a left-wise coiled shell, and compared the expression patterns with the wild-type (dextral, right-wise coiled shell) strain of the same species [9].

## Methods

### Animal handling

Animal handling followed the guidelines for animal experiments of the University of Tokyo.

### Animals

Individuals of *P. vulgata* were collected in Shaldon, Devon, UK, and *N. fuscoviridis* in Tateyama, Chiba, Japan. The strains of *L. stagnalis* were reared in tap water in the laboratory. We cultured the dextral strain and sinistral mutant strain of *L. stagnalis* (derived from Shinshu University). Throughout the year, these organisms lay eggs in capsules coated with jelly. Methods of egg collection and culturing followed those in the previous studies on *N. fuscoviridis* and *L. stagnalis*[10,11].

### RNA extraction, cDNA synthesis, and gene cloning

We used the mantle tissues of *P. vulgata, N. fuscoviridis,* and *L. stagnalis* for RNA extraction. The mantle tissues were cut off into two parts, left and right. The total RNA was extracted (ISOGEN; Nippon Gene Co. Ltd, Tokyo, Japan), and cDNA synthesis was performed (ReverTra Ace; Toyobo, Osaka, Japan) in accordance with the product protocols. We isolated elongation factor 1 alpha (EF-1α) sequences from *P. vulgata* and *N. fuscoviridis* using degenerate primers designed for Mollusca [12] (see Additional file 1: Figure S1). We used EF-1α-specific primers for *L. stagnalis* as reported previously [13]. After purification of PCR products using a commercial kit (Gel Extraction Kit; Qiagen Science Inc., Valencia, CA, USA), amplicons were ligated into a vector (pGEM-T Easy Vector; Promega Corp., Madison, WI, USA) using a DNA ligation kit (Promega Corp.), and then transformed to DH5α competent cells (Toyobo).

### Quantitative reverse transcriptase PCR

Because it is difficult to analyze gene expression patterns in adult specimens using whole-mount *in situ* hybridization, we performed quantitative reverse transcription (qRT)-PCR instead. We designed qRT-PCR primers using the software Primer 3 (see Additional file 2: Table S1). Relative quantification of total RNA was performed using a commercial solution (SsoFast EvaGreen supermix with low ROX; Bio-Rad Laboratories, Inc., Hercules, CA, USA) and a real-time PCR system (Step One; Applied Biosystems, Foster City, CA, USA). The production of gene-specific products was confirmed by checking their melting curves at the end of qRT-PCR reactions. Data acquisition and analysis were performed (ABI Step One™ software version 2.0; Applied Biosystems). Baselines and thresholds for Ct were set automatically. Quantifications of the target genes were performed by the relative standard curve method. To normalize the quantification of the target gene (*dpp*) expression, we used the housekeeping gene, EF-1α.

### Whole-mount *in situ* hybridization

We performed *in situ* hybridization as described previously for amphioxus [14], except for the following changes in the conditions to make it suitable for molluscan embryos. We fixed the *L. stagnalis* embryos with 4% paraformaldehyde in MTSTr (50 mmol/l PIPES-KOH pH 6.9, 25 mmol/l EGTA, 150 mmol/l KCl, 25 mmol/l MgCl_2_, and 0.1% Triton X-100) [15]. For the other limpet, *P. vulgata,* embryos were fixed with MEMPFA-T (0.1 mol/l MOPS pH 7.4, 2 mmol/l EGTA, 1 mmol/l MgSO_4_, 4% paraformaldehyde, and 0.1% Tween 20) [6] overnight at 4°C.

### Western blotting

Proteins in the mantle tissues were extracted (ISOGEN; Nippon Gene, Tokyo, Japan) in accordance with the manufacturer’s protocol, and were dissolved afterwards in buffer (NuPAGE LDS Sample Buffer; Life Technologies, Corp., Carlsbad, CA, USA). We carried out electrophoresis using 20 μg protein samples on pre-cast polyacrylamide gels with a linear gradient of 4 to 20% (Bio-Rad, Laboratories, Inc., Hercules, CA, USA), and transferred the separated proteins to nitrocellulose membranes. Blocking was performed overnight using 3% BSA in Tris-buffered saline with Tween (TBS-T: 25 mmol/l Tris HCl pH 7.4, 137 mmol/l NaCl, 2.7 mmol/l KCl, and 0.1% Tween-20) at 4°C. Immunodetection was performed using phosphorylated SMAD1/5/8 polyclonal antibody (#9516; Cell Signaling Technology, Danvers, MA, USA) and SMAD1/5/8 polyclonal antibody (sc-6031-R; Santa Cruz Biotechnology, Santa Cruz, CA, USA) at 1:1000 dilution in a commercial solution (Can Get Signal solution 1; Toyobo Co. Ltd, Osaka, Japan). After overnight incubation with the primary antibody at 4°C, the membrane was washed three times in TBS-T, and incubated overnight at 4°C with horseradish peroxidase (HRP)-labeled anti-rabbit antibodies (Thermo Fisher Scientific Inc., Rockford, IL, USA) that were diluted 1:2000 in a commercial solution (Can Get Signal solution 2; Toyobo,). After washing the membrane three times in TBS-T, it was incubated with a western blotting detection reagent (ECL Prime; GE Healthcare Life Sciences, Little Chalfont, Buckinghamshire UK). The enhanced chemiluminescence signals were detected with a lumino image analyzer (LAS-1000 Plus; Fuji Film, Japan). We measured these signals using ImageJ software (version 1.46.)

### Statistical analysis

The Wilcoxon–Mann–Whitney test was performed using the statistical software R (version 2.7.1) to evaluate the significant differences in expression levels between the left and right parts of the mantle tissue. *P*<0.05 was considered significant.

## Results

**Figure 2:**
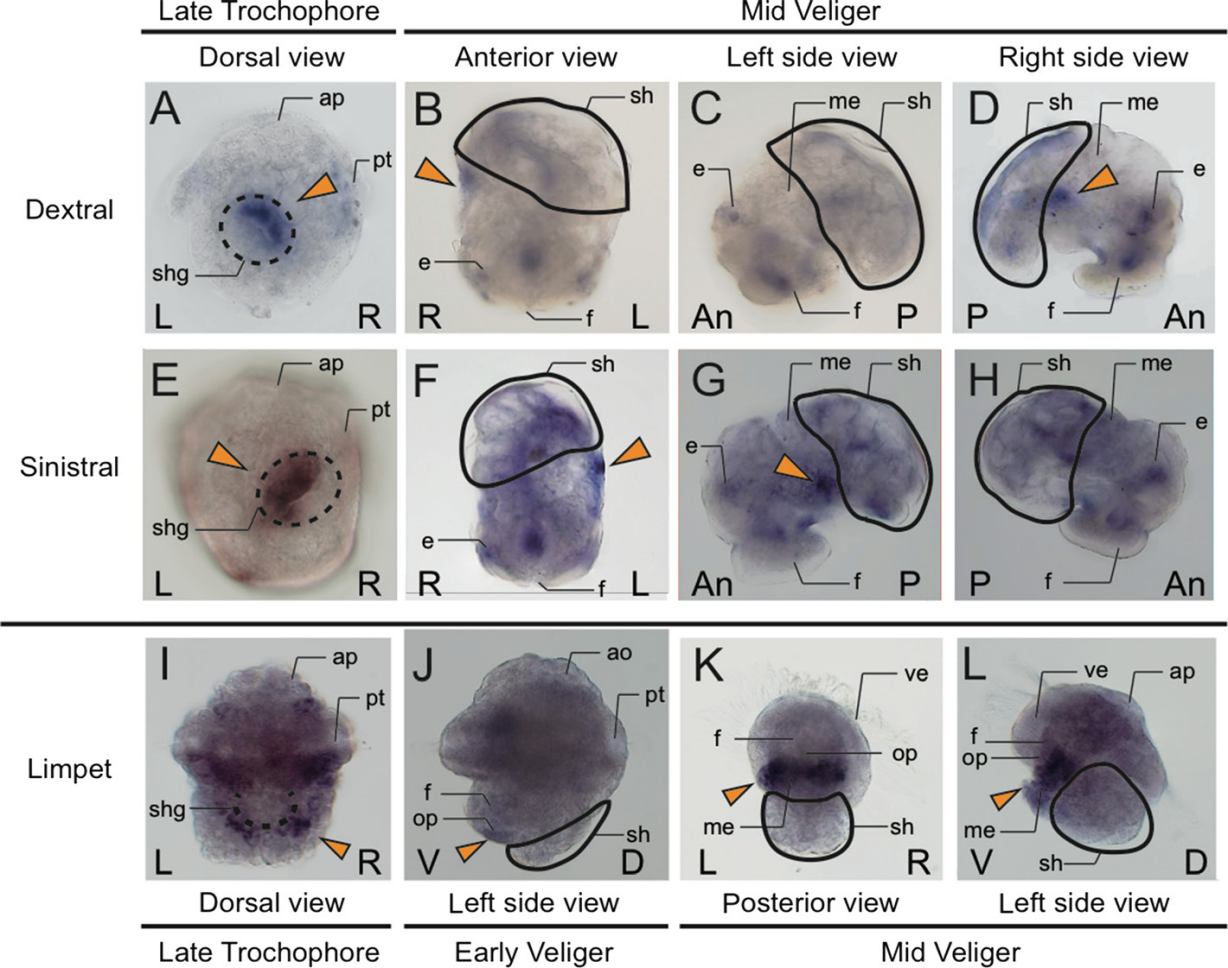
**Expression patterns of *dpp *in the trochophore and veliger stages.** Expression patterns of *dpp* in (**A**, **E**, **I**) the trochophore and **(B**–**D**, **F**-**H**, **J**-**L**) veliger stages of the pond snail *Lymnaea stagnalis*, which has a coiled shell (**A**-**D**) dextral strain; (**E**-**H**) sinistral strain) and (**I**-**L**) the limpet *Patella vulgata*. (**A**, **E**, **I**) Shell gland (dorsal) view; (**B**, **F**) anterior view; (**C**, **G**, **J**, **K**) left side view; (**D**, **H**) right side view; (**K**) posterior view. (**A**, **E**, **I**) Broken black lines indicate the shell gland, and arrowheads indicate the *dpp* expression. (**A**-**D**, **F**-**H**, **J**-**K**) Black lines indicate the shell. (**A**–**H**) Expression of *dpp* is asymmetric in the shell gland or mantle edge in late trochophore and mid-veliger sages. (**I**, **K**, **L**) Expression of *dpp* is symmetric in late trochophore and mid-veliger stages. (**J**) *dpp* is expressed in the operculum gland. An, anterior; ap, apical plate; d, dorsal; e, eye; f, foot; L, left; m, mouth; me, mantle edge; op, operculum; P, posterior; Pt, prototroch; R, right; sh, shell; shg, shell gland opening; V, ventral; ve, velum.

In the trochophore of the sinistral mutants of *L. stagnalis*, *dpp* is expressed in the left half of the shell gland, mirroring the pattern of the dextral strain, which shows expression of *dpp* only in the right half of the shell (Figure 2A,E). Such asymmetrical expression patterns were seen in the veliger stage also: *dpp* is expressed in the mantle edge as a small spot in the right side only or the left side only in *L. stagnalis* (dextral strain, Figure 2B-D; sinistral strain, F-H). By contrast, *dpp* shows a symmetrical expression pattern in the limpet *P. vulgata*, with *dpp* being expressed circularly around the shell gland at the late trochophore stage (Figure 2I) [6]. In the early veliger stage, *dpp* ceases to be expressed in the shell field and is expressed in the operculum gland (Figure 2) [8]. However, *dpp* shows symmetric expression in the mantle edge again at the mid-veliger stage (Figure 2J and K).

**Figure 3:**
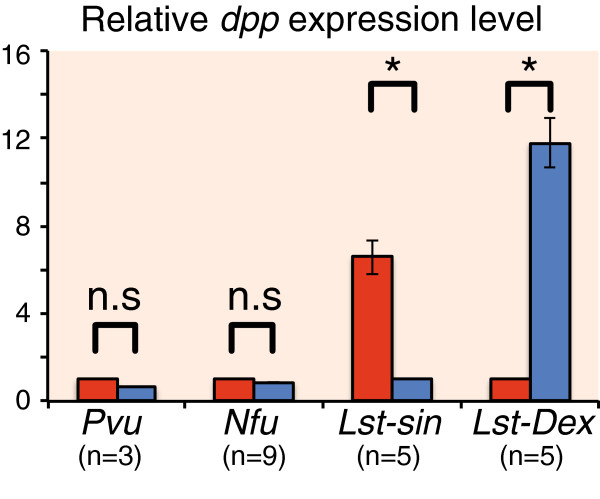
**Expression levels of *dpp *transcripts in adult mantle edge tissue.** Comparison of the levels of *dpp* transcripts between the left and right sides of the mantle tissue by quantitative reverse transcription (RT)-PCR analysis using EF-1α transcripts as reference. In the non-coiled shelled limpets, *Patella vulgata* and *Nipponacmea fuscoviridis*, *dpp* expression levels were not different between left and right sides of mantle tissues. By contrast, in the coiled-shell snail *Lymnaea stagnalis* (dextral and sinistral)*, dpp* expression levels were significantly different (asymmetric) (Wilcoxon–Mann–Whitney test; *P*<0.05). Gene expression levels were standardized by dividing the values by those of the left side (*P. vulgate*, *N. fuscoviridis,* and the dextral strain of *L. stagnalis*), or by those of the right side (sinistral strain of *L. stagnalis*). Error bars represent standard deviations.

We then compared the *dpp* expression levels between left and right sides of the mantle edges using qRT-PCR analysis in the three gastropod species. We again found different expression patterns between the coiled and non-coiled shell of the gastropods, consistent with the gene expression patterns described above. In the two limpets *P. vulgata* and *N. fuscoviridis*, whose shells are non-coiled, there was no difference in the *dpp* expression levels between tissue samples taken from the left and the right sides of their mantle edge (Figure 3). By contrast, there was asymmetric *dpp* expression between the left and right sides was seen in the coiled shell *L. stagnalis*; *dpp* expression is higher in the right side of the mantle edge of the wild-type dextral line individuals, and higher in the left side mantle edge in the sinistral mutant individuals (Figure 3).

**Figure 4:**
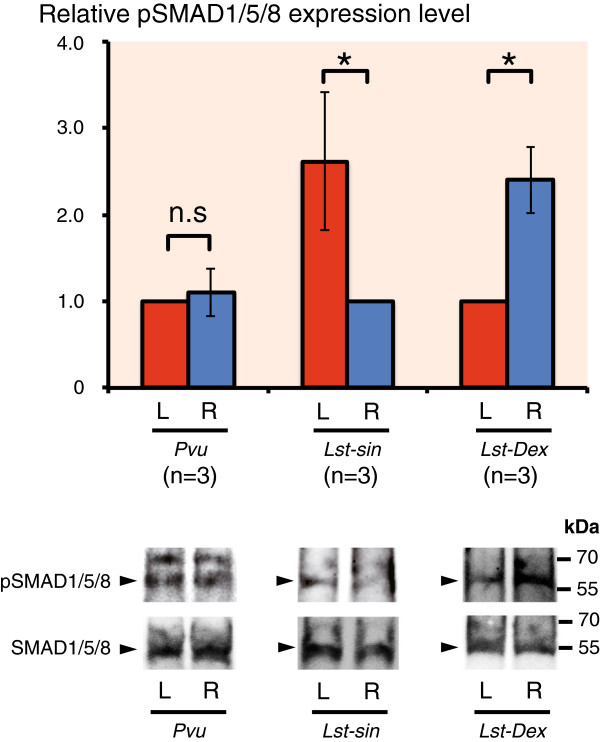
**Expression levels of pSMAD1/5/8 in adult mantle edge tissues.** Comparisons of the levels of pSMAD1/5/8 between left and right sides of the mantle tissue by western blotting. In the non-coiled shelled limpet *Patella vulgata,* pSMAD1/5/8 expression levels were not different between left and right sides of the mantle edges. By contrast, in the coiled-shell snail *Lymnaea stagnalis* (dextral and sinistral)*,* pSMAD1/5/8 expression levels were significantly different (asymmetric) (paired t-test; *P*<0.05). Expression levels were standardized by dividing the values by those of the left side (*P. vulgata*, *Nipponacmea fuscoviridis,* and the dextral strain of *L. stagnalis*), or by those of the right side (sinistral strain of *L. stagnalis*). Error bars represent standard deviations.

To confirm the presence of the Dpp gradient in the growing mantle tissues, we compared expression levels of phosphorylated SMAD1/5/8 (pSMAD1/5/8) in the mantle edges using western blotting. In the non-coiled limpet *P. vulgata*, there was no significant difference in pSMAD1/5/8 expression between left and the right sides of the mantle edge (Figure 4), whereas there was asymmetric expression of pSMAD1/5/8 in the coiled shelled snail *L. stagnalis* (Figure 4). These results indicate that a Dpp signal gradient indeed exists in the mantle edge of the coiled-shell snail, whereas Dpp signals are distributed symmetrically in the non-coiled-shell limpet.

## Discussion

In the field of theoretical morphology of biological shapes, coiling shells have drawn considerable interest for many years. Rice [16] provided a theoretical model based on the idea that the animal must keep a constant gradient of shell growth rate between the outer and inner edge (the gradient) to produce a coiling shell. This idea has been incorporated in many recent models for shell growth (for example, Hammer *et al.*[17]. Urdy *et al*. [18]. By contrast, the molecular basis of shell coiling is poorly understood to date. Probably a morphogen-like gradient substance exists, but no candidate for such a concentration gradient has yet been identified. Our results suggest that the left–right gradient of the Dpp protein (caused by a left–right asymmetric expression of the *dpp* gene) could be the most likely candidate for the gradient in shell coiling, as discussed for some previous mathematical models [16-18].

**Figure 5:**
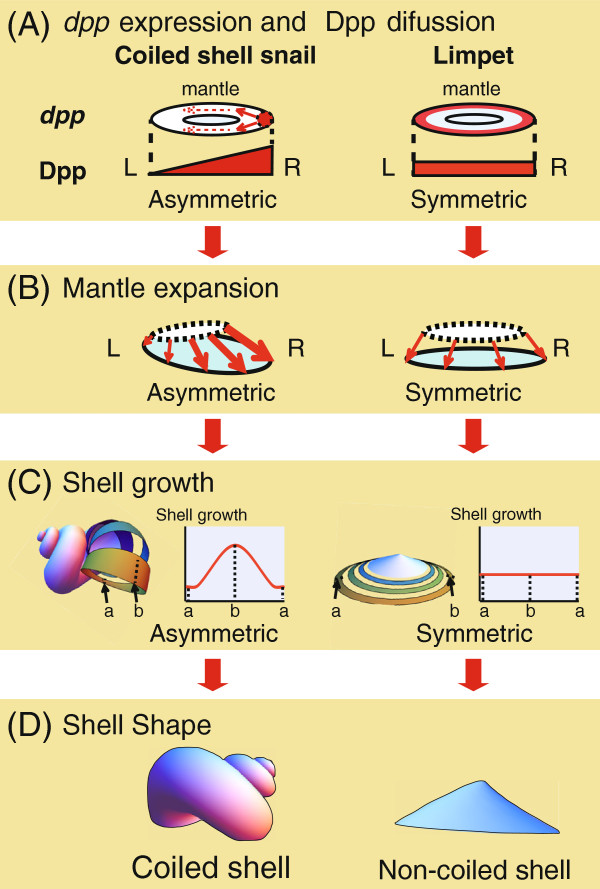
**A molecular hypothesis of shell coiling in Gastropoda. (A)** In a snail with a coiled shell, *dpp* (red) is expressed asymmetrically in the mantle, and Dpp diffusion causes an asymmetric concentration gradient in the mantle. **(B)** Asymmetric mantle expansion is induced by asymmetric Dpp localization, because Dpp controls cell proliferation in the mantle [8]. **(C, D)** As a result of the asymmetric mantle expansion, non-uniform shell growth occurs, and produces a coiled shell. By contrast, in the limpets, a non-coiled shell is formed because the lack of expression of *dpp* in the mantle results in symmetric mantle expansion and shell growth. L, left; R, right.

In this study, we found that in the coiled-shell snail *L. stagnalis*, *dpp* is expressed in the local spot of the left or right side mantle edge that corresponds with the shell-coiling direction at the veliger stage, and continues being expressed asymmetrically until the adult stage (Figure 2A-H; Figure 3). By contrast, in the limpets, *dpp* continues to be expressed symmetrically from the late trochophore stage to the adult stage (Figure 2I,K,L; Figure 3). Furthermore, we found by western blotting using anti-phosphorylated SMAD1/5/8 antibodies that Dpp signals are indeed distributed asymmetrically in the mantle edge in the coiled-shell snail and symmetrically in the non-coiled-shell limpet (Figure 4). In the fruit fly, Dpp works as a morphogen during wing development, spreading through the target point and forming a concentration gradient that provides positional information [19]. Rogulja *et al*. [20] further showed that Dpp triggers cell division, and the division activity correlates positively with the concentration of Dpp gradient. Hashimoto *et al*. [8] suggested that in gastropods, Dpp might function by triggering the regulation of cell division in the mantle during shell formation. The cells of the mantle edge secrete shell-matrix proteins, and these proteins are transferred to the outer edge of the shell and mineralized with CaCO_3_. Therefore, if cells rapidly proliferate, more cells can secrete shell-matrix proteins in any one unit of time. We thus propose that during coiled-shell development, Dpp acts as a trigger for an asymmetric cell proliferation, by producing a concentration gradient in the mantle from one spot of expression, and diffuses to the other side of the mantle (Figure 5A). The Dpp gradient might then cause several different reaction thresholds, which in turn induce different levels of cell proliferation along the aperture (Figure 5B). These different levels of cell division might then cause an asymmetric aperture expansion, causing a non-uniform shell growth (Figure 5C) and resulting in a coiled shell (Figure 5D). Constant asymmetric expression of *dpp,* and thus a constant presence of the gradient until the veliger and adult stage of the snail, ensures the constant coiling during shell growth. Meanwhile, in the non-coiled-shelled limpets, symmetric aperture expansion and shell growth occurs because *dpp* is expressed symmetrically in the shell gland and the mantle edge, causing uniform cell division (Figure 2, Figure 3, Figure 4, Figure 5).

A recent report [11] of functional analysis of Dpp in *L. stagnalis* supports this hypothetical mechanism of shell coiling. When the embryos were treated with a Dpp signal inhibitor (dorsomorphin) at the trochophore and veliger stages, the juvenile shells showed a cone-like form rather than a normal coiled form [11]. These results indicated that Dpp signals induce differences in shell growth rates around the aperture by their gradient. The molecular results presented here support this mathematical models for shell growth [16-18].

**Figure 6:**
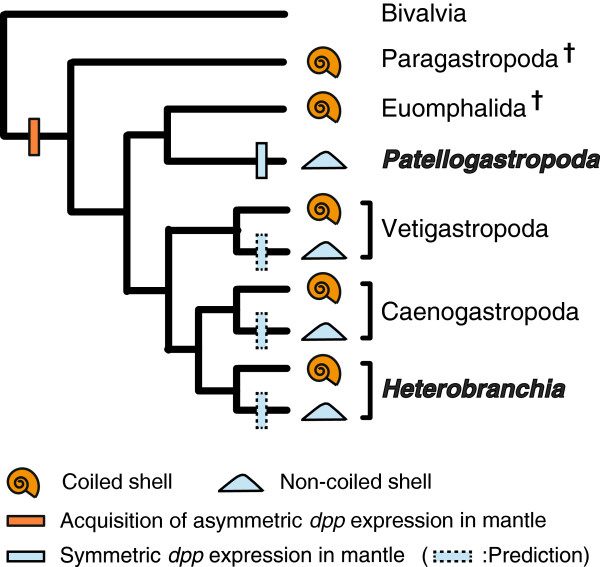
**Evolutionary hypothesis of the shell-coiling mechanism in Gastropoda.** The most recent common ancestor of Gastropoda acquired the asymmetric *dpp* expression pathway in the mantle at one stage (orange line). Later, the Patellogastropoda lost this pathway and the non-coiled shell shape evolved in this group (blue line). Moreover, other species with non-coiled shells in Vetigastropoda, Caenogastropoda or Heterobranchia most likely evolved like Patellogastropoda (broken blue lines).

The molecular developmental insights into shell coiling reported here also explain how shell coiling was lost several times during the evolution of gastropods. Although it is difficult to infer the ancestral shell shape (coiled or non-coiled shell), previous phylogenetic studies showed that the non-coiled-shelled gastropod Patellogastropoda is placed as the sister group to the rest of extant gastropods (Figure 1; Figure 6). However, considering the fossil record, Paragastropoda that have coiled shells are possibly the most recent common ancestor of gastropods [1], hence suggesting that the coiled-shell feature is probably synplesiomorphy and the non-coiled shell shape has evolved independently several times in gastropods (Figure 1; Figure 6) [1,2]. Our current results suggest that the loss of coiling might have happened relatively easily, by losing the asymmetric expression of *dpp* (or its upstream regulators) in the shell gland at the trochophore stage, and leading to symmetric *dpp* expression n the veliger and adult stages. Further investigations are needed to understand the molecular mechanisms of shell formation and evolution, because the process of shell development is very complex. However, the new insight provided by the current study into *dpp* expression patterns in the mantle edge, not only in the early developmental stages but also in later stages, is the key basis for understanding how various shell shapes evolved and are formed in gastropods.

In this study, we found that continuous expression of *dpp* in the mantle edge until the adult stage might explain the mechanism of these two variations in gastropod shell shapes, that is, the coiled and the non-coiled shapes. However, because in this study we used only patellogastropod species (*P. vulgata* and *N. fuscoviridis*), further molecular studies of the species other than those of the Patellogastropoda, such as those from other non-coiled-shell snails are needed in order to be able to infer a decisive conclusion about the evolution of shell-coiling loss in gastropods (Figure 1).

## Conclusion

We found crucial differences in *dpp* expression patterns between non-coiled-shell limpets and coiled-shell gastropods with a dextral or a sinistral shell, not only in the early developmental stages but also in the late stages. By cross-referencing with previous functional analyses of *dpp* in gastropods and other animals [8,11,19,20] and previous mathematical models ([16-18], we suggest a hypothesis of shell coiling based on the presence of a Dpp gradient. We hypothesize that Dpp induces mantle expansion, corresponding to the pattern of the concentration gradient of the Dpp morphogen (Figure 5). This hypothesis provides plausible biological grounds for previously published mathematical models of shell formation [16-18]. Our results also suggest a molecular explanation for he shell-coiling mechanism in gastropods, and thus provide robust preliminary information to answer the question about how the diverse gastropod shell shapes evolved.

## Abbreviations

Dpp: *Decapentaplegic*; EF-1α: Elongation factor 1 alpha; Pvu: *Patella vulgata*; Nfu: *Nipponacmea fuscoviridis*; Lst: *Lymnaea stagnalis*; Dex: Dextral; Sin: Sinistral

## Competing interests

The authors declare that they have no competing interests.

## Authors’ contributions

KS conducted most of the experiments and led the project. MI and DS helped with the Whole mount *in situ* hybridization experiments in gastropods embryo. TA and EG gave the dextral and sinistral strains of L. stagnalis. MI, DS, IS, TK and KE conceived the study, and participated in the design and coordination of the project. All authors participated in writing the manuscript drafts, and then discussed and approved the final version of the manuscript.

## Supplementary Material

Additional file 1: Figure S1 Phylogenetic relationships of elongation factor 1 alpha. Sequence Alignment was performed by MAFFT (http://mafft.cbrc.jp/alignment/server/index.html). Maximum Likelihood (ML) phylogenetic analysis was done using MEGAv5.0 with 100 bootstrap replications. Bootstrap supports below 50% are not shown. *Gallus gallus* (L00677.1), *Xenopus laevis* (NM_001101761.1), *Drosophila melanogaster* (X06869.1), *Hediste japonica* (AB003702), *Lamellibrachia sp.* (AB003721), *Allolobophora sp.* (AB003714), *Myxobdella sinaensis* (AB003716), *Capitella sp.* (AB003706), *Calyptogena soyoae* (AB003719), *Aplysia juliana* (DQ916605.1), *Batillus cornutus* (AB003720), *Haliotis rufenscens* (DQ087488.1), *Lottia jamaicensis* (FJ977772.1). Click here for file

Additional file 2: Table S1 Sequences of primers used in this study. Click here for file
