# Supplementary figures and images for: Left-right asymmetric expression of dpp in the mantle of gastropods correlates with asymmetric shell coiling
Source: EvoDevo. 2013 May 28;4:15. doi: 10.1186/2041-9139-4-15 (PMC3680195; doi:10.1186/2041-9139-4-15)

Supplementary Figure S1.

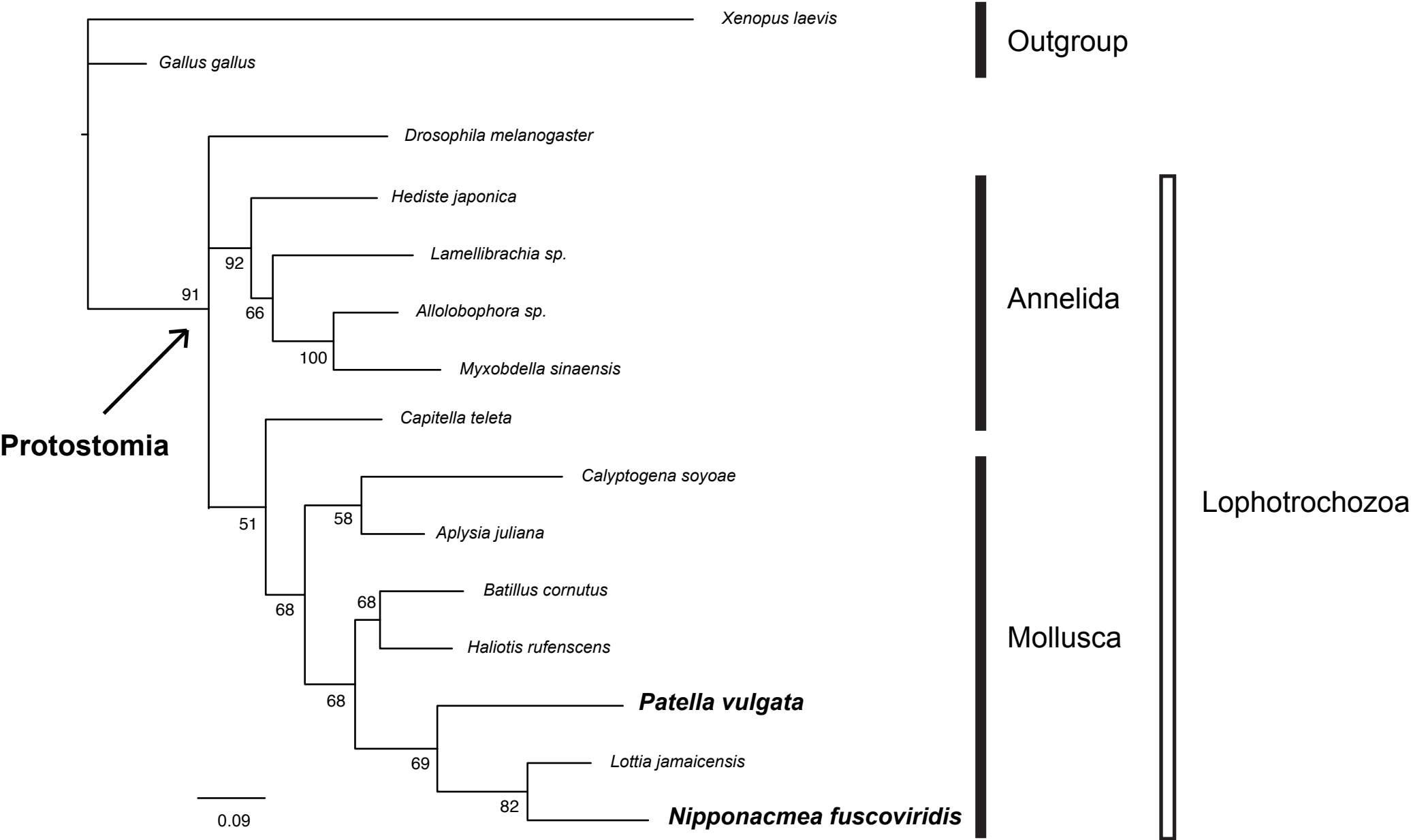

Supplement: Additional file 1: Figure S1 — Phylogenetic relationships of elongation factor 1 alpha. Sequence Alignment was performed by MAFFT (http://mafft.cbrc.jp/alignment/server/index.html). Maximum Likelihood (ML) phylogenetic analysis was done using MEGAv5.0 with 100 bootstrap replications. Bootstrap supports below 50% are not shown. Gallus gallus (L00677.1), Xenopus laevis (NM_001101761.1), Drosophila melanogaster (X06869.1), Hediste japonica (AB003702), Lamellibrachia sp. (AB003721), Allolobophora sp. (AB003714), Myxobdella sinaensis (AB003716), Capitella sp. (AB003706), Calyptogena soyoae (AB003719), Aplysia juliana (DQ916605.1), Batillus cornutus (AB003720), Haliotis rufenscens (DQ087488.1), Lottia jamaicensis (FJ977772.1). [file 2041-9139-4-15-S1.pdf]
